# Establishment of reference intervals for complete blood count in times of COVID-19 and vaccination

**DOI:** 10.11613/BM.2023.020701

**Published:** 2023-04-15

**Authors:** Claudia Mendieta-Gutiérrez, Selene Chávez-González, Brenda Ivonne Rodríguez-Romero, Jazmín Anahí Sánchez-Garrido, Arturo Figueroa-Gómez, Jesús Bernabé Licona-Vela, Arturo Cuauhtémoc Juárez-Pérez, Alejandro Cabello-López, Guadalupe Aguilar-Madrid, Carmina Jiménez-Ramírez

**Affiliations:** 1 Clinical Analysis Laboratory, Traumatology Hospital “Dr. Victorio de la Fuente Narváez”, High Specialty Medical Unit, Mexican Social Security Institute (IMSS), Mexico City, México; 2 Clinical Analysis Laboratory, Regional Zone Hospital No.24, IMSS, Mexico City, México; 3 Occupational Health Research Unit, National Medical Center “Siglo XXI”, IMSS, Mexico City, México; 4 Department of Public Health, Faculty of Medicine, Universidad Nacional Autónoma de México (UNAM), Mexico City, Mexico

**Keywords:** reference intervals, complete blood count, COVID-19, vaccine

## Abstract

**Introduction:**

COVID-19 and vaccination may affect some parameters of the complete blood count (CBC). The aim of this study was to determine reference intervals (RI) of CBC in healthy population with different COVID-19 and vaccination backgrounds and compare them with those established previously.

**Materials and methods:**

A cross-sectional study was conducted in donors who attended the Traumatology Hospital “Dr. Victorio de la Fuente Narváez” (HTVFN) from June to September 2021. Reference intervals were established using the non-parametric method on Sysmex XN-1000. For differences between groups with different COVID-19 and vaccination backgrounds, non-parametric tests were used.

**Results:**

The RI were established in 156 men and 128 women. Haemoglobin (Hb), haematocrit (Hct), red blood cells (RBC), platelets (Plt), mean platelets volume (MPV), monocytes and relative neutrophils were higher in men than women (P < 0.001). The percentiles of Hb, Hct, RBC, MPV and relative monocytes showed higher values; Plt, white blood cells (WBC), lymphocytes, monocytes, neutrophils, eosinophils and absolute basophils, the 2.5th was higher and the 97.5th was lower; for lymphocytes and relative neutrophils, both percentiles had a trend toward lower values, compared to previous RI. Differences between groups with different COVID-19 and vaccination backgrounds, in lymphocytes (P = 0.038), neutrophils (P = 0.017) and eosinophils (P = 0.018) in men; Hct (P = 0.014), RDW (P = 0.023) in women and MPV (P = 0.001) in both, were not considered pathological.

**Conclusions:**

The RI for the CBC were established in a Mestizo-Mexican population with different COVID-19 and vaccination backgrounds, so should be updated and validated in different hospitals close to the HTVFN that use the same analyser.

## Introduction

The reference intervals (RI) of the complete blood count (CBC) reported by the clinical laboratory are essential for the diagnosis and treatment of patients. The Clinical and Laboratory Standards Institute (CLSI) and the International Federation of Clinical Chemistry and Laboratory Medicine (IFCC) have published guidelines for establishing them (*1*, *2*). The clinical laboratory of the Hospital of Traumatology ‘Dr Victorio de la Fuente Narváez’ (HTFVN), of the Mexican Social Security Institute (IMSS), currently applies the RI established in 2015 for the CBC of a healthy Mestizo-Mexican population who donated blood that year.

In February 2020, the Coronavirus Disease 2019 (COVID-19) pandemic reached Mexico, and in December 2020, vaccination of the public for COVID-19 began (*3*, *4*). Therefore, the population who donate blood were individuals who may or may not have a COVID-19 background and have already recovered and who may or may not be vaccinated.

After recovery from COVID-19, a leucocytosis, lymphocytosis, or lower haemoglobin and eosinophils are presented (*5*, *6*). After vaccination, a decrease in platelets (Plt) are reported (*7*). To our knowledge, follow-up studies of haematological alterations in patients recovered from COVID-19 and vaccinated are scarce, so it is unknown if the possible haematological sequelae would modify the RI established before pandemic and vaccination.

After the start of the pandemic, there were few published studies where RI of the CBC had been determined, but the populations included had not been affected by the COVID-19 pandemic and the vaccination (*8*, *9*). The aim of this study was to determine the RI of the CBC in blood donors with different COVID-19 and vaccination backgrounds, following the CLSI guidelines to compare our RI with the previous RI and to determine the differences in the COVID-19 and vaccination backgrounds.

## Materials and methods

### Subjects

A descriptive cross-sectional study was conducted on donors who attended the blood bank service of HTVFN (in process of accreditation by Joint Commission International) in Mexico City from June to September 2021. The Mexican Official Standard (NOM) 007-SSA3-2011 states that all laboratory reports must include the RI of each analyte by sex and age group (*10*). Donor candidates were previously instructed to present with a minimum of 8 hours of fasting and a rest of at least 8 hours, previous low-fat diet, no strenuous exercise. During venous blood sampling on the forearm (duration time approximately 4 minute), donation candidates remained seated. We included men and women who were accepted as donors in accordance with the guidelines of the NOM 253-SSA1-2012, which establishes the requirements for the provision of human blood and its components for therapeutic purposes: their age ranged from 18 to 65 years, no alcohol, caffeine or tobacco use, non-pregnant and non-breastfeeding women, no oral contraceptive and/or aspirin treatment, who weighed more than 50 kg and who were considered healthy by questionnaire and medical examination, and who, if they had a COVID-19 background, their donation was postponed by 14 days after the disappearance of symptoms (*11*, *12*). Only donors who had been vaccinated with attenuated virus vaccines, whole attenuated or inactivated virus vaccines or if they did not know the type of vaccine administered, should defer their donation for 14 days and individuals who had received mRNA vaccines, non-replicating viral vector, or protein subunit vaccines, did not need to defer donation (*4*). Donors who decided not to participate in this study at the end of their donation process, who had lipaemic and/or haemolysed samples, who presented obesity and donors with surnames that were not of Spanish origin and with less than three generations living in the country were excluded. All donors who agreed to participate signed an informed consent. The present study was approved by the ethics and research committee of the HTVFN (institutional registration number R-2021-3401005).

### Methods

In this study, all donors underwent a medical examination that included measurement of blood pressure, height, heart rate, history of illness, smoking, drinking habits, *etc.* If they were considered clinically healthy, they were accepted as blood donors. Subsequently, a 5 mL sample of venous blood was collected from the forearm in vacutainer tubes with dipotassium salt of ethylenediaminetetraacetic acid (K2-EDTA) as an anticoagulant (Beckton Dickinson, Franklin Lakes, USA), all samples were analysed within 30 minutes of collection on the Sysmex XN-1000 analyser (Sysmex Corporation, Kobe, Japan).

Compliance with the low, medium and high internal quality control (QC) (XN CHECK, Sysmex Corporation, Kobe, Japan), was checked daily on the analyser prior to sample processing. In addition, the HTVFN laboratory participates in a National Programme for External Quality Assessment (NPEQA) (JAR Quality SA de CV) accredited by the International Organization for Standardization (ISO) and International Electrotechnical Commission (IEC) (ISO/IEC 17043). Assay precision, accuracy, linearity and carryover were evaluated on the analyser (Supplementary material).

Blood samples were taken daily until a minimum sample size of 120 donors *per* sex was reached; this sample size was established by the CLSI guidelines to determine the RI with a 90% confidence interval (CI) (*1*). All socio-demographic and COVID-19 and vaccination backgrounds data were collected by the Hexabank software (Abbott Laboratories, Tharsis-it Information Technology), which was used in the medical interview. The RI calculations were done according to sex. Subsequently, men and women were divided into the following groups: recovered COVID-19 individuals, vaccinated individuals, recovered COVID-19 and vaccinated, for comparison with the group of individuals without COVID-19 and vaccination backgrounds (control group).

### Statistical analysis

Normality of the data distribution was determined by the Shapiro-Wilk test. The RI were determined in accordance with CLSI guidelines, using the 2.5th and 97.5th percentiles with a 90% CI (1,10). Outliers were determined by the Tukey test. The Mann-Whitney U test was used to compare the groups with different COVID-19 and vaccination backgrounds and the control group. For comparison between groups, the Kruskal-Wallis test was used. A significance level of less than 0.05 was considered statistically significant. Stata 14 SE (StataCorp LLC, Texas, USA) was used for analyses of the data.

## Results

The RI of the CBC was determined in 284 donors, 156 men and 128 women. The data are shown in Table 1.

**Table 1:** Socio-demographic characteristics of the blood donors

**Variables**	**Total (N = 284)**	**Men (N = 156)**	**Women (N = 128)**
Residence			
Mexico City	116	56	60
State of Mexico	135	77	58
Other	33	23	10
Age (years)*	35 (18-63)	35 (18-63)	35 (19-63)
COVID-19 Background			
No	240	128	112
Yes	44	28	16
Time after COVID-19 (months)*	11 (1 - 21)	12 (1 - 21)	9 (3 - 13)
Vaccination Background			
No	139	67	72
Yes	145	89	56
Type of vaccine			
BNT162b2 (Pfizer/BioNTech, USA and Germany)	18	7	11
Oxford‐AstraZeneca COVID‐19 vaccine AZD1222 (University Oxford & AstraZeneca)	69	45	24
Ad5-nCoV (CanSino Biologics, China)	4	3	1
mRNA-1273 (ModernaTx, Inc and Sinopharm, USA)	1	1	-
CoronaVac (Sinovac Biotech, China)	20	13	7
Sputnik V (Gamaleya Res Inst, Russia)	26	17	9
Unknown	7	3	4
Time after Vaccine (days)*	45 (3 - 270)	45 (3 - 270)	60 (7 - 180)
COVID-19 and Vaccination Background			
COVID-19: No; Vaccine: No	124	58	66
COVID-19: Yes; Vaccine: No	15	9	6
COVID-19: No; Vaccine: Yes	116	70	46
COVID-19: Yes; Vaccine: No	29	19	10
*Age, time after COVID-19 and time after vaccine, are represented as median (minimum-maximum).			

Tables 2 and 3 shows the RI established for men and women separately and the RI previously used at the HTVFN. For both sexes, the 2.5th and 97.5th percentiles calculated for haemoglobin (Hb), haematocrit (Hct), red blood cells (RBC), mean platelet volume (MPV) and relative monocytes showed a trend towards higher values compared to those recorded previously. For Plt, white blood cells (WBC), eosinophils and relative basophils, lymphocytes, monocytes, neutrophils, eosinophils and absolute basophils, the 2.5th percentile was higher and the 97.5th percentile was lower, causing the RI to be narrower. For lymphocytes and relative neutrophils, both percentiles had a trend toward lower values.

**Table 2:** Established RI for complete blood count obtained in men blood donors and its comparison with previous RI

**Haematological parameters**	**Calculated RI (N = 156)**				**Previous RI**		**Differences in percentiles (%)**	
**Percentile**				**Percentile**	
	**2.5th**	**97.5th**	**Lower 90% CI**	**Upper 90% CI**	**2.5th**	**97.5th**	**2.5th**	**97.5th**
Hb (g/L)	156	185	153 - 157	180 - 185	138	177	+ 11.3	+ 4.1
Hct (L/L)	0.463	0.536	0.460 - 0.467	0.529 - 0.539	0.420	0.530	+ 9.3	+ 1.1
RBC (x10^12^/L)	5.03	6.36	4.97 - 5.15	6.22 - 6.40	4.30	5.70	+ 14.5	+ 10.4
RDW (%)	11.5	13.9	11.3 - 11.7	13.7 - 14.0	-	-	-	-
Plt (x10^9^/L)	175	388	168 - 194	367 - 394	150	450	+ 14.3	- 16.0
MPV (fL)	8.9	12.4	8.4 - 9.0	12.2 - 12.6	6.8	10.4	+ 23.6	+ 16.1
WBC (x10^9^/L)	4.9	10.9	4.6 - 5.2	10.3 - 11.3	4.5	11.0	+ 8.2	- 0.9
Lymphocytes (%)	21	48	15 - 23	47 - 52	25	50	- 17.9	- 3.5
Monocytes (%)	5	11	5 - 6	11 - 12	2	10	+ 63.0	+ 9.9
Neutrophils (%)	38.9	68.0	36.2-41.8	66.2-71.6	50	80	- 28.5	- 17.6
Eosinophils (%)	0	4	0 - 1	3 - 4	0	5	+ 100.0	- 42.9
Basophils (%)	0	1	0	1 - 2	0	2	+ 100.0	- 53.8
NRBC (%)	0	0	-	-	-	-	-	-
IG (%)	0	1	0	1	-	-	-	-
Lymphocytes (x10^9^/L)	1.45	3.74	1.27 - 1.52	3.52 - 3.96	1.0	5.0	+ 31.0	- 33.7
Monocytes (x10^9^/L)	0.35	0.92	0.27 - 0.38	0.86 - 0.96	0.0	1.0	+ 100.0	- 8.7
Neutrophils (x10^9^/L)	2.20	6.70	1.49 - 2.34	6.00 - 6.83	2.0	8.0	+ 9.1	- 19.4
Eosinophils (x10^9^/L)	0.03	0.30	0.02 - 0.04	0.26 - 0.33	0.0	0.40	+ 100.0	- 33.3
Basophiles (x10^9^/L)	0.012	0.092	0.010 - 0.020	0.090 - 0.100	0.0	0.4	+ 100.0	- 334
NRBC (x10^9^/L)	0.0	0.0	-	-	-	-	-	-
IG (x10^9^/L)	0.010	0.051	0.001 - 0.012	0.050 - 0.053	-	-	-	-
RI – Reference intervals. CI - Confidence interval. Hb – Haemoglobin. Hct – Haematocrit. RBC – Red blood cells. RDW - Red cell distribution width. Plt – Platelets. MPV - Mean platelet volume. WBC - White blood cells. NRBC - Nucleated red blood cell. IG - Immature granulocytes. Differences between the established and previous RI are shown in the percentile difference column expressed as a percentage and with (+) if the change was higher than the previous RI or (-) if it was lower.								

**Table 3:** Established RI for complete blood count obtained in women blood donors and its comparison with previous RI

**Haematological parameters**	**Calculated RI (N = 128)**					**Previous RI**		**Differences in percentiles (%)**	
	**Percentile**					**Percentile**			
	**2.5th**	**97.5th**		**Lower 90% CI**	**Upper 90% CI**	**2.5th**	**97.5th**	**2.5th**	**97.5th**
Hb (g/L)	132	165		131 - 135	161 - 168	119	157	+ 9.8	+ 4.8
Hct (L/L)	0.406	0.521		0.400 - 0.415	0.503 - 0.523	0.360	0.470	+ 11.3	+ 9.8
RBC (x10^12^/L)	4.35	5.75		4.29 - 4.45	5.52 - 5.96	3.80	5.00	+ 12.6	+ 13.0
RDW (%)	11.5	14.1		11.4 - 11.8	13.8 - 14.6	-	-		
Plt (x10^9^/L)	185	386		177 - 206	366 - 412	150	450	+ 18.9	- 16.6
MPV (fL)	8.8	12.9		8.4 - 8.9	12.5 - 13.1	6.8	10.4	+ 22.7	+ 19.4
WBC (x10^9^/L)	4.9	10.7		3.7 - 5.2	10.3 - 11.1	4.5	11.0	+ 8.2	- 2.8
Lymphocytes (%)	19	47		17 - 22	45 - 50	25	50	- 33.0	- 6.6
Monocytes (%)	4	10		4 - 5	10 - 11	2	10	+ 53.5	+ 1.0
Neutrophils (%)	44	70		42 - 46	68 - 72	50	80	- 14.2	- 14.9
Eosinophils (%)	0	3		0 - 1	3 - 4	0	5	+ 100.0	- 51.5
Basophils (%)	0	1		0	1	0	2	+ 100.0	- 73.9
NRBC (%)	0	0		-	-	-	-	-	-
IG (%)	0	1		0	1	-	-	-	-
Lymphocytes (x10^9^/L)	1.28	3.75		1.07 - 1.49	3.50 - 3.84	1.0	5.0	+ 21.9	- 33.3
Neutrophils (x10^9^/L)	2.17	6.80		1.25 - 2.27	6.56 - 7.34	2.0	8.0	+ 7.8	- 17.6
Monocytes (x10^9^/L)	0.27	0.80		0.21 - 0.31	0.75 - 0.91	0.0	1.0	+ 100.0	- 25.0
Eosinophils (x10^9^/L)	0.03	0.26		0.02 - 0.04	0.22 - 0.27	0.0	0.40	+ 100.0	- 53.8
Basophiles (x10^9^/L)	0.020	0.080		0.017 - 0.023	0.072 - 0.088	0.0	0.4	+ 100.0	- 400.0
NRBC (x10^9^/L)	0.0	0.0		-	-	-	-	-	-
IG (x10^9^/L)	0.012	0.045		0.008 - 0.016	0.041 - 0.049	-	-	-	-
RI – Reference intervals. CI - Confidence interval. Hb – Haemoglobin. Hct – Haematocrit. RBC – Red blood cells. RDW - Red cell distribution width. Plt – Platelets. MPV - Mean platelet volume. WBC - White blood cells. NRBC - Nucleated red blood cell. IG - Immature granulocytes. Differences between the established and previous RI are shown in the percentile difference column expressed as a percentage and with (+) if the change was higher than the previous RI or (-) if it was lower.									

Table 4 shows significantly higher Hb, Hct, RBC, Plt, MPV, relative and absolute monocytes and relative neutrophils in men than in women (P < 0.001).

**Table 4:** Differences in complete blood count determined by sex

**Haematological parameters**	**Men (N = 156)**	**Women (N = 128)**	**P**
Hb (g/L)	168 ± 6	147 ± 8	< 0.001
Hct (L/L)	0.502 (0.490 - 0.511)	0.454 (0.430 - 0.474)	< 0.001
RBC (x10^12^/L)	5.66 ± 0.30	4.98 ± 0.33	< 0.001
RDW (%)	12.5 (12.2 - 13.0)	12.6 (12.1 - 13.1)	0.517
Plt (x10^9^/L)	256 (226 - 294)	284 (249 - 318)	< 0.001
MPV (fL)	10.1 (9.6 - 10.9)	10.8 (10.0 - 11.6)	< 0.001
WBC (x10^9^/L)	7.3 (6.3 - 8.3)	7.3 (6.3 - 8.4)	0.889
Lymphocytes (%)	34 ± 7	33 ± 7	0.302
Monocytes (%)	8 (7 - 9)	7 (6 - 8)	< 0.001
Neutrophils (%)	54 ± 8	57 ± 7	0.002
Eosinophils (%)	2 (1 - 2)	2 (1 - 2)	0.786
Basophils (%)	1 (0 - 1)	1 (0 - 1)	0.149
IG (%)	0.3 ± 0.1	0.3 ± 0.1	0.119
Lymphocytes (x10^9^/L)	2.4 ± 0.6	2.4 ± 0.6	0.454
Monocytes (x10^9^/L)	0.58 (0.48 - 0.69)	0.50 (0.43 - 0.63)	< 0.001
Neutrophils (x10^9^/L)	3.95 (3.17 - 4.64)	4.04 (3.33 - 5.02)	0.218
Eosinophils (x10^9^/L)	0.13 (0.08 - 0.18)	0.12 (0.08 - 0.16)	0.296
Basophiles (x10^9^/L)	0.04 (0.03 - 0.06)	0.04 (0.03 - 0.05)	0.177
IG (x10^9^/L)	0.02 (0.01 - 0.03)	0.02 (0.01 - 0.03)	0.072
Variables are presented as median (interquartile range). Hb – Haemoglobin. Hct – Haematocrit. RBC - Red blood cells. RDW - Red blood cell distribution width. Plt – Platelets. MPV - Mean platelet volume. WBC - White blood cells. NRBC - Nucleated Rred blood cell. IG - Immature granulocytes. Hb, RBC, lymphocytes (%), neutrophils (%), IG (%) and lymphocytes (x10^9^/L) are shown as mean ± standard deviation and the differences between men and women were determined by the Student t-test. For the rest of the variables, differences were determined by Mann-Whitney U test. P < 0.05 was considered statistically significant.			

Table 5 and 6 show the differences in CBC between the groups with different COVID-19 and vaccination backgrounds and control group in men and women, respectively. Despite the differences found between the groups, the values obtained were not considered pathological.

**Table 5:** Complete blood count in men donors with different COVID-19 and vaccination background

**Haematological parameters**	**COVID-19: No** **Vaccine: No** **(N = 58)**	**COVID-19: Yes** **Vaccine: No** **(N = 9)**	**COVID-19: No** **Vaccine: Yes** **(N = 70)**	**COVID-19: Yes** **Vaccine: Yes** **(N = 19)**	**P^†^**
Hb (g/L)	168 (164-171)	169 (163-172)	168 (162 - 173)	169 (165 - 171)	0.930
Hct (L/L)	0.506 (0.494 - 0.513)	0.502 (0.477 - 0.506)	0.500 (0.488 - 0.508)	0.515 (0.488 - 0.514)	0.473
RBC (x10^12^/L)	5.76 (5.56 - 5.86)	5.63 (5.43 - 6.00)	5.60 (5.45 - 5.77)	5.72 (5.57 - 5.89)	0.145
RDW (%)	12.4 (12.1 - 12.9)	12.4 (12.0 - 12.6)	12.7 (12.1 - 13.2)	12.8 (12.1 - 13.2)	0.055
			0.017‡		
Plt (x10^9^/L)	256 (226 - 291)	288 (248 - 294)	256 (224 - 303)	249 (230 - 279)	0.765
MPV (fL)	10.6 (9.9 - 11.8)	10.4 (10.2 - 10.7)	10.1 (9.6 - 10.8)	10.0 (9.3 - 10.1)	0.001
			0.007^‡^	0.001^§^	
WBC (x10^9^/L)	7.5 (5.9 - 8.0)	6.7 (6.6 - 6.9)	7.3 (6.5 - 8.5)	7.1 (6.1 - 8.9)	0.368
Lymphocytes (%)	34 (28 - 39)	34 (31 - 39)	33 (28 - 36)	39 (33 - 42)	0.038
					0.004^ǁ^
Monocytes (%)	8 (7 - 10)	9 (7 - 9)	8 (7 - 9)	8 (7 - 9)	0.319
Neutrophils (%)	53 (48 - 59)	56 (50 - 60)	56 (52 - 61)	50 (45 - 55)	0.017
Eosinophils (%)	2 (1 - 3)	1 (1 - 1)	2 (1 - 2)	2 (1 - 3)	0.018
		0.023^¶^	0.037^‡^		
Basophils (%)	1 (1 - 1)	1 (1 - 1)	1 (0 - 1)	1 (1 - 1)	0.228
NRBC (%)	0 (0 - 0)	0 (0 - 0)	0 (0 - 0)	0 (0 - 0)	-
IG (%)	0.33 (0.20 - 0.40)	0.33 (0.30 - 0.40)	0.34 (0.20 - 0.40)	0.33 (0.20 - 0.40)	0.941
Lymphocytes (x10^9^/L)	2.45 (2.01 - 2.93)	2.28 (2.11 - 2.34)	2.43 (2.04 - 2.87)	2.58 (2.19 - 3.03)	0.454
Monocytes (x10^9^/L)	0.59 (0.47 - 0.69)	0.59 (0.44 - 0.60)	0.57 (0.49 - 0.70)	0.56 (0.48 - 0.61)	0.341
Neutrophils (x10^9^/L)	3.93 (2.94 - 4.59)	3.85 (2.95 - 4.48)	4.08 (3.57 - 4.94)	3.57 (2.90 - 4.47)	0.218
Eosinophils (x10^9^/L)	0.14 (0.08 - 0.20)	0.09 (0.06 - 0.10)	0.13 (0.09 - 0.16)	0.14 (0.09 - 0.18)	0.194
Basophiles (x10^9^/L)	0.04 (0.03 - 0.07)	0.05 (0.03 - 0.06)	0.04 (0.03 - 0.06)	0.05 (0.03 - 0.06)	0.612
NRBC (x10^9^/L)	0.0 (0.0 - 0.0)	0.0 (0.0 -0.0)	0.0 (0.0 - 0.0)	0.0 (0.0 - 0.0)	-
IG (x10^9^/L)	0.02 (0.01 - 0.03)	0.03 (0.02 - 0.04)	0.03 (0.03 - 0.04)	0.02 (0.01 - 0.04)	0.520
Variables are presented as median (interquartile range). Hb – Haemoglobin. Hct – Haematocrit. RBC - Red blood cells. RDW - Red blood cell distribution width. Plt – Platelets. MPV - Mean platelet volume. WBC - White blood cells. NRBC - Nucleated blood cell. IG - Immature granulocytes. The group without COVID-19 and vaccination backgrounds was considered as the control group. The differences between groups with different COVID-19 and vaccination backgrounds and the control group, were compared with the Mann-Whitney U test. For comparison between groups, the Kruskal-Wallis test was used. ^†^Comparison between groups. ^‡^Comparison between group COVID-19: no, vaccine: yes, with control group. ^§^Comparison between group COVID-19: yes, vaccine: yes, with control group. ^ǁ^Comparison between group COVID-19: no, vaccine: yes, with group COVID-19: yes, vaccine: yes. ^¶^Comparison between group COVID: yes, vaccine no, with control group. P < 0.05 was considered statistically significant.					

**Table 6:** Complete blood count in women donors with different COVID-19 and vaccination backgrounds

**Haematological parameters**	**COVID-19: No** **Vaccine: No** **(N = 66)**	**COVID-19: Yes** **Vaccine: No** **(N = 6)**	**COVID-19: No** **Vaccine: Yes** **(N = 46)**	**COVID-19: Yes** **Vaccine: Yes** **(N = 10)**	**P^†^**
Hb (g/L)	149 (143 - 154)	146 (142 - 152)	145 (139 - 152)	143 (136 - 150)	0.095
Hct (L/L)	0.463 (0.439 - 0.481)	0.439 (0.422 - 0.468)	0.438 (0.424 - 0.462)	0.434 (0.423 - 0.467)	0.014
			0.002^‡^		
RBC (x10^12^/L)^12^/L)	5.02 (4.80 - 5.27)	4.78 (4.62 - 5.04)	4.97 (4.75 - 5.29)	4.90 (4.72 - 5.09)	0.481
RDW (%)	12.4 (12.1 - 13.0)	12.1 (11.9 - 12.4)	12.9 (12.5 - 13.3)	12.7 (12.1 - 13.3)	0.023
			0.007^‡^		
Plt (x10^9^/L)	281 (244 - 310)	260 (238 - 333)	288 (257 - 315)	307 (259 - 323)	0.591
MPV (fL)	11.4 (10.3 - 12.0)	10.6 (9.3 - 11.1)	10.2 (9.7 - 11.0)	10.4 (9.4 - 10.7)	0.001
			0.001^‡^	0.014^§^	
WBC (x10^9^/L)	7.2 (6.5 - 8.4)	6.3 (5.5 - 7.1)	7.3 (6.3 - 8.7)	8.0 (6.4 - 8.9)	0.481
Lymphocytes (%)	33 (29 - 37)	34 (27 - 38)	33 (30 - 35)	37 (31 - 39)	0.428
Monocytes (%)	7 (6 - 8)	7 (7 - 7)	6 (6 - 8)	6 (6 - 8)	0.303
Neutrophils (%)	57 (52 - 62)	59 (55 - 60)	57 (55 - 60)	54 (50 - 61)	0.694
Eosinophils (%)	2 (1 - 2)	2 (1 - 2)	2 (1 - 2)	2 (1 - 2)	0.732
Basophils (%)	1 (0 - 1)	1 (0 - 1)	1 (0 - 1)	1 (1 - 1)	0.992
NRBC (%)	0 (0 - 0)	0 (0 - 0)	0 (0 - 0)	0 (0 - 0)	-
IG (%)	0.30 (0.20 - 0.40)	0.30 (0.30 - 0.40)	0.30 (0.20 - 0.40)	0.30 (0.20 - 0.40)	0.725
Lymphocytes (x10^9^/L)	2.41 (2.00 - 2.76)	2.19 (1.77 - 2.43)	2.40 (2.11 - 2.82)	2.46 (2.28 - 2.97)	0.507
Monocytes (x10^9^/L)	0.53 (0.45 - 0.63)	0.42 (0.38 - 0.47)	0.49 (0.40 - 0.60)	0.52 (0.40 - 0.63)	0.114
Neutrophils (x10^9^/L)	4.05 (3.27 - 4.92)	3.47 (3.33 - 3.87)	4.14 (3.45 - 5.10)	4.03 (3.29 - 5.05)	0.690
Eosinophils (x10^9^/L)	0.12 (0.08 - 0.15)	0.10 (0.07 - 0.11)	0.12 (0.09 - 0.16)	0.15 (0.10 - 0.18)	0.645
Basophiles (x10^9^/L)	0.04 (0.03 - 0.06)	0.03 (0.03 - 0.05)	0.04 (0.03 - 0.05)	0.04 (0.04 - 0.05)	0.744
NRBC (x10^9^/L)	0.0 (0.0 - 0.0)	0.0 (0.0 - 0.0)	0.0 (0.0 - 0.0)	0.0 (0.0 - 0.0)	-
IG (x10^9^/L)	0.02 (0.01 - 0.03)	0.02 (0.02 - 0.03)	0.02 (0.01 - 0.03)	0.02 (0.02 - 0.03)	0.640
Variables are presented as median (interquartile range). Hb – Haemoglobin. Hct – Haematocrit. RBC - Red blood cells. RDW - Red blood cell distribution width. Plt – Platelets. MPV - Mean platelet volume. WBC - White blood cells. NRBC - Nucleated blood cell. IG - Immature granulocytes. The group without COVID-19 and vaccination backgrounds was considered as the control group. The differences between groups with different COVID-19 and vaccination backgrounds and the control group, were compared with the Mann-Whitney U test. For comparison between groups, the Kruskal-Wallis test was used. ^†^Comparison between groups. ^‡^Comparison between group COVID-19: no, vaccine: yes, with control group. ^§^Comparison between group COVID-19: yes, vaccine: yes, with control group. P < 0.05 was considered statistically significant.					

## Discussion

Accurate and update RI for CBC are important to establishing the diagnosis and treatment for patients. In this study, we established a new RI for the CBC. The previously used RI were established in 200 donors (100 men and 100 women), selected with the same inclusion and exclusion criteria as the donors included in this study. The percentiles of Hb, Hct, RBC, MPV and relative monocytes showed higher values compared to previous RI. These differences could be attributed to different socio-demographic characteristics of Mexico City (main financial, political and cultural centre of the country) and the State of Mexico (surrounds México City). Donors previously were mainly from Mexico City (60-70%). In this study, the donors came mainly from two areas with different altitude, State of Mexico with average 2300 m and Mexico City, specifically from the Alvaro Obregón municipality (average 3200 m) (*13*, *14*). Donors previously were mainly from different municipalities with an average altitude of 2240 m (*13*). Higher altitude generates a state of hypoxia that stimulates erythropoiesis (*15*). The RI for lymphocytes, monocytes, neutrophils, eosinophils and basophils changed considerably from the previous RI. This may be attributed to medical assessment because although the inclusion and exclusion criteria of the NOM-253-SSA1 for donors were applied, the recent guidelines established by the National Transfusion Centre were also integrated, so that the medical evaluation was stricter, and no possible inflammatory process went unnoticed (*4*, *11*, *12*). The current RI will have an impact because it will reduce the number of patients admitted to surgery with lower Hb and platelet concentrations that were considered safe with the previous RI, reduce the number of blood products transfused during surgery and allow for proper diagnosis of anaemia, thrombocytopenia, sepsis, and inflammatory diseases. The RI reported by Díaz-Piedra *et al.* differs our RI because, although sample size was higher, included individuals under 20 years of age, health status is unknown, and another analyser was used (*16*). Reference intervals vary according to the population in which they were established (*1*, *17*).

In Mexico, the Mestizo-Mexican population who is currently donating blood has been affected, in some cases, by previous infections of COVID-19 and by the vaccination process. Relative eosinophils in men with a COVID-19 background were lower, and this difference was significant, but not considered pathological. An improvement in eosinophil counts and the presence of leucocytosis and lymphocytosis has been reported in individuals who have recovered from COVID-19, after 4 weeks (*5*, *18*, *19*). Our results partially agree with those reported for eosinophils but not for the rest of the cells. It is possible that this is due to donors with a COVID-19 background, donating blood between 6 and 12 months after discharge. In addition, it should also be considered that the number of donors in this group was smaller.

The differences found in the vaccinated group of relative neutrophils and eosinophils in men; Hct in women and RDW in both sexes, may attributed to the immune response, which is affected by sex and was triggered by vaccine administration (*20*-*24*). Higher RDW but within normal ranges, as we found, would indicate a response to infection, but in our study, it could have been a response to the vaccine mimicking infection (*20*, *25*).

It was not possible to analyse the influence of the time interval after vaccination because the vaccination programme varied in the type of vaccine and the date of application, according to age group and geographical area, so there were donors who could donate the day after vaccination, donors who had to be deferred and others who, because of their age or the geographical area in which they lived, were more than 270 days after vaccination when they came forward to donate.

We found lower MPV in vaccinated donors and in donors who, in addition to being vaccinated, had a COVID-19 background. Thrombotic thrombocytopenia can occur after administration of certain vaccines (*26*). However, there are no reports of MPV alterations in vaccinated individuals. It is important to mention that different vaccines are administered in Mexico, with different mechanisms to generate immunity (*4*, *27*).

Although significant differences were found between the groups with different COVID-19 and vaccination backgrounds, this study did not show values that were considered abnormal (leucocytosis, eosinopenia, thrombocytopenia, *etc.*), but it is possible that these differences have caused a trend towards higher or lower values of the RI of the CBC which were calculated before the pandemic and vaccinations. However, further studies are needed to explore possible long-term haematological alterations in COVID-19 recovered individuals as well as vaccinated individuals and how these conditions may or may not modify previously established RI in a population.

The limitations of this study lie in the fact that although the minimum sample size required to determine the RI was obtained in a single range of age, when we categorise the different groups by COVID-19 and vaccination backgrounds, some groups had small sample sizes, and vaccination backgrounds were only obtained by questionnaire, so there may have been a bias among donors to deny their disease background or vaccination for fear of being rejected as donors. In addition to the fact that different vaccines have been administered to the population, some with different schedules and, unfortunately, we do not know the number of donors who had COVID-19 and were asymptomatic.

In conclusion, the RI for the CBC were established in a Mestizo-Mexican population by sex, with different COVID-19 and vaccination backgrounds, so should be updated in the respective laboratory report. The RI obtained can be validated in different hospitals close to the HTVFN that use the same analyser, as the population they serve has the same characteristics as the donors with whom they were established.
